# Supplementary material for: Predicting dominant terrestrial biomes at a global scale using machine learning algorithms, climate variable indices, and extreme event indices
Source: PLoS One. 2026 Feb 26;21(2):e0324107. doi: 10.1371/journal.pone.0324107 (PMC12944746; doi:10.1371/journal.pone.0324107)

S2 Fig. Histograms of average monthly climate indices (*AveI*, 16 variables). Red bars: averages for 1970-2000; Blue bars averages for 2061-2080.

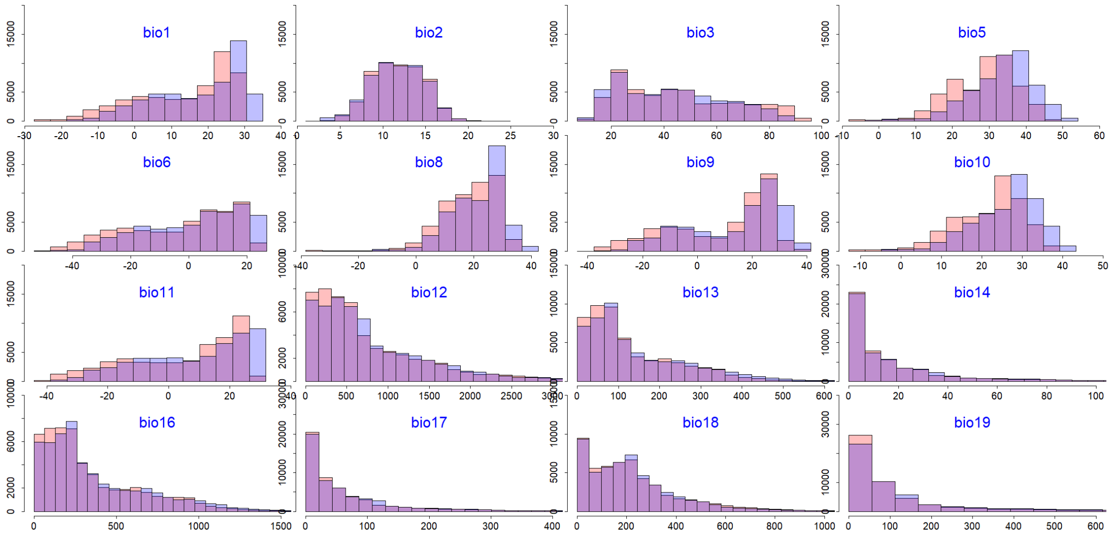

Supplement: S2 Fig — Red bars: averages for 1970–2000; Blue bars averages for 2061–2080. (PDF) [file pone.0324107.s002.pdf]
