# Supplementary material for: Predicting dominant terrestrial biomes at a global scale using machine learning algorithms, climate variable indices, and extreme event indices
Source: PLoS One. 2026 Feb 26;21(2):e0324107. doi: 10.1371/journal.pone.0324107 (PMC12944746; doi:10.1371/journal.pone.0324107)

S4 Fig. Simulated potential natural vegetation (PNV) under current climatic conditions using the RF model. Four sets of climate data were used for training and simulation: (a) *Ave*, (b) *AveI*, (c) *Ave* + *CEI*, (d) *AveI* + *CEI*, (e) *Ave* + CEI_{part}, and (f) *AveI* + CEI_{part}.

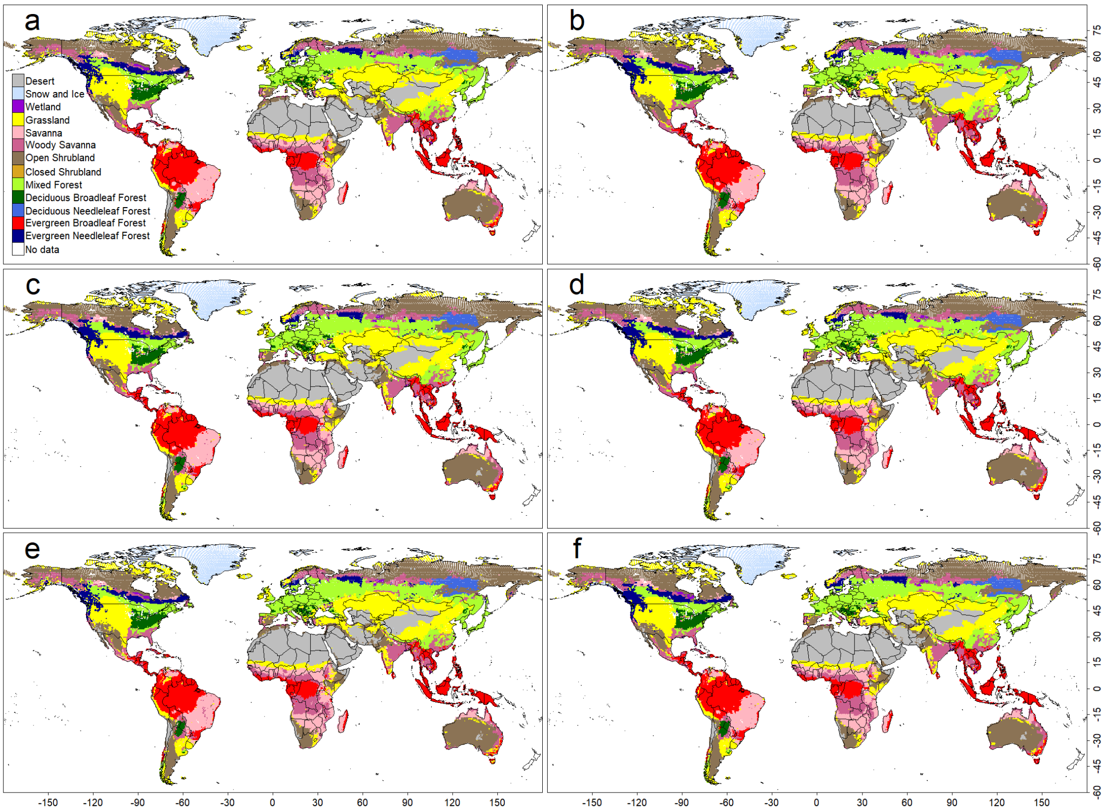

Supplement: S4 Fig — Four sets of climate data were used for training and simulation: (a) Ave, (b) AveI, (c) Ave + CEI, (d) AveI + CEI, (e) Ave + CEIpart, and (f) AveI + CEIpart. (PDF) [file pone.0324107.s004.pdf]
