# Supplementary material for: Predicting dominant terrestrial biomes at a global scale using machine learning algorithms, climate variable indices, and extreme event indices
Source: PLoS One. 2026 Feb 26;21(2):e0324107. doi: 10.1371/journal.pone.0324107 (PMC12944746; doi:10.1371/journal.pone.0324107)

S7 Fig. Simulated PNV under current climatic conditions using the CNN model. The same experimental setup as in S4 Fig. was used.

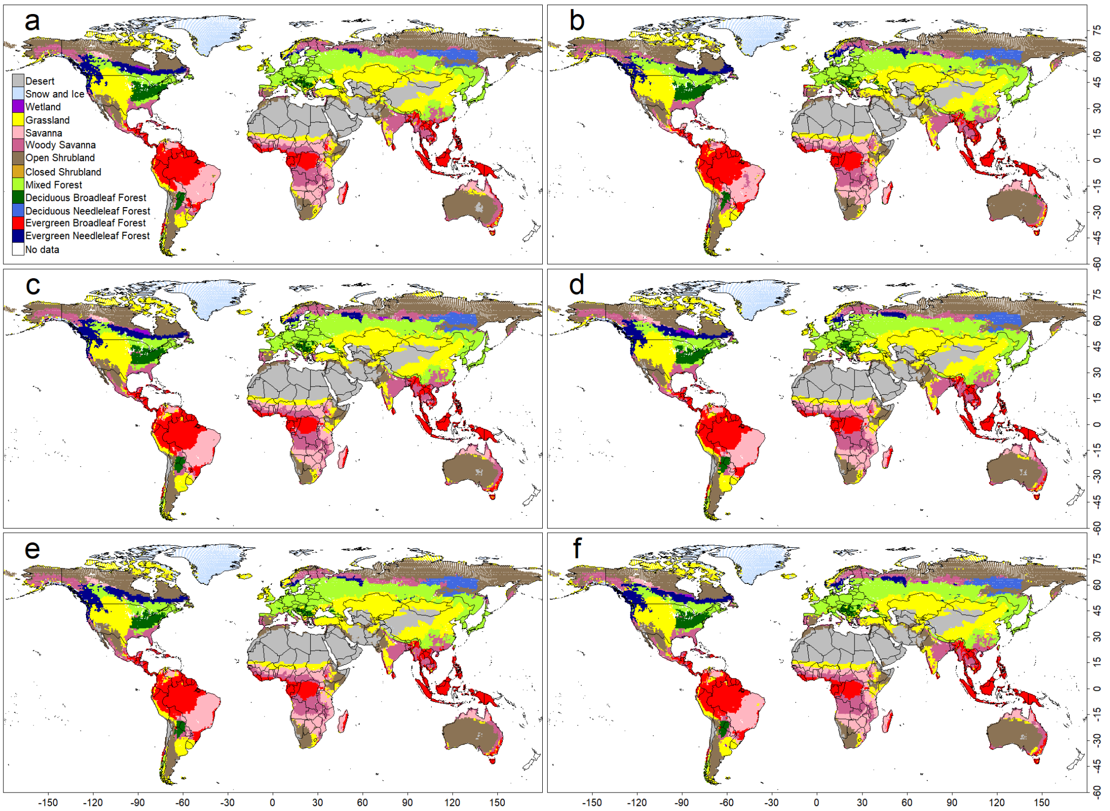

Supplement: S7 Fig — The same experimental setup as in S4 Fig. was used. (PDF) [file pone.0324107.s007.pdf]
