# Supplementary material for: Predicting dominant terrestrial biomes at a global scale using machine learning algorithms, climate variable indices, and extreme event indices
Source: PLoS One. 2026 Feb 26;21(2):e0324107. doi: 10.1371/journal.pone.0324107 (PMC12944746; doi:10.1371/journal.pone.0324107)
Supplement: S1 Table — From the IGBP classification, three human-mediated classifications (Croplands, Cropland/Natural Vegetation Mosaics, and Urban and Built-Up Lands) and Water Bodies were neglected. Descriptions were based on Loveland and Belward. (PDF) [file pone.0324107.s012.pdf]

S1 Table. Potential Natural Vegetation (PNV) classes used in the modelings. From the IGBP classification, three human-mediated classifications (Croplands, Cropland/Natural Vegetation Mosaics, and Urban and Built-Up Lands) and Water Bodies were neglected. Descriptions were based on Loveland and Belward (1).

Name	Description	Prevalence
Evergreen Needleleaf Forests	Canopy cover of needle leaf trees >60% and height >2m. Evergreen needle-leaf trees dominate.	2.4%
Evergreen Broadleaf Forests	Canopy cover of broad leaf trees >60% and height >2m. Evergreen broadleaf trees dominate.	11.2%
Deciduous Needleleaf Forests	Woody vegetation cover >60% and height >2m. Deciduous needle-leaf trees (larch) dominate.	0.9%
Deciduous Broadleaf Forests	Woody vegetation cover >60% and height >2m. Deciduous broadleaf trees dominate.	2.0%
Mixed Forests	Woody vegetation cover >60% and height >2m. Consists of interspersed mixtures or mosaics of the other four forest cover types. None of the forest types exceeds 60% of the landscape.	9.5%
Closed Shrublands	Lands with woody vegetation <2m in height and with shrub canopy cover >60%. The shrub foliage can be either deciduous or evergreen.	0.2%
Open Shrublands	Lands with woody vegetation <2m in height and with shrub canopy cover between 10-60%. The shrub foliage can be either evergreen or deciduous.	16.8%
Woody Savannas	Lands with herbaceous and other understory plants and with forest canopy cover between 30-60%. The forest cover height is >2m.	11.5%
Savannas	Lands with herbaceous and other understory plants and with forest canopy cover between 10-30%. The forest cover height is >2m.	9.3%
Grasslands	Lands dominated by grass. Tree and shrub cover is <10%.	17.8%
Wetlands	Lands with a permanent mixture of water and grass or woody vegetation cover extensive areas. The foliage can be present in either salt, brackish, or fresh water.	0.5%
Snow and Ice	Lands under snow/ice cover most of the year.	2.3%
Barren	Lands with exposed soil, sand, or rocks and <10% vegetative cover throughout the year.	15.5%

Reference

1. Loveland TR, Belward AS. The International Geosphere Biosphere Programme Data and Information System global land cover data set (DISCover). Acta Astronaut. 1997;41(4-10):681-9. doi: 10.1016/s0094-5765(98)00050-2. PubMed PMID: WOS:000074499200050.
