# Supplementary material for: Predicting dominant terrestrial biomes at a global scale using machine learning algorithms, climate variable indices, and extreme event indices
Source: PLoS One. 2026 Feb 26;21(2):e0324107. doi: 10.1371/journal.pone.0324107 (PMC12944746; doi:10.1371/journal.pone.0324107)
Supplement: S2 Table — Columns represent the actual classes, while rows represent the predicted class. This matrix is based on the test grid only, with a total of 392,230 predictions from 10 independent trials (39,223 grids×10 test). Shaded diagonal cells indicate correct classifications. Each cell shows the count (top) and column-wise percentage (bottom) within the actual class. (PDF) [file pone.0324107.s013.pdf]

S2 Table. Confusion matrix for biome classification using the *AVE* climate dataset and the RF model. Columns represent the actual classes, while rows represent the predicted class. This matrix is based on the test grid only, with a total of 392,230 predictions from 10 independent trials (39,223 grids × 10 test). Shaded diagonal cells indicate correct classifications. Each cell shows the count (top) and column-wise percentage (bottom) within the actual class.

Predicted Class (columns); Actual Class (rows)

	A	B	C	D	E	F	G	H	I	J	K	L	M
A	6147<br>65.3%	0<br>0.0%	0<br>0.0%	42<br>0.4%	1569<br>16.7%	1<br>0.0%	94<br>1.0%	678<br>7.2%	106<br>1.1%	750<br>8.0%	11<br>0.1%	20<br>0.2%	1<br>0.0%
B	0<br>0.0%	39832<br>90.6%	0<br>0.0%	21<br>0.0%	236<br>0.5%	0<br>0.0%	22<br>0.1%	2118<br>4.8%	1375<br>3.1%	349<br>0.8%	34<br>0.1%	0<br>0.0%	0<br>0.0%
C	0<br>0.0%	0<br>0.0%	2580<br>77.0%	8<br>0.2%	274<br>8.2%	0<br>0.0%	220<br>6.6%	209<br>6.2%	8<br>0.2%	31<br>0.9%	19<br>0.6%	0<br>0.0%	0<br>0.0%
D	5<br>0.1%	28<br>0.4%	0<br>0.0%	4991<br>62.8%	1777<br>22.3%	6<br>0.1%	50<br>0.6%	372<br>4.7%	259<br>3.3%	453<br>5.7%	6<br>0.1%	0<br>0.0%	4<br>0.1%
E	813<br>2.2%	303<br>0.8%	94<br>0.3%	994<br>2.7%	30394<br>81.6%	7<br>0.0%	143<br>0.4%	2201<br>5.9%	64<br>0.2%	2178<br>5.8%	48<br>0.1%	2<br>0.0%	2<br>0.0%
F	1<br>0.1%	21<br>2.5%	0<br>0.0%	12<br>1.4%	36<br>4.3%	61<br>7.3%	248<br>29.8%	122<br>14.7%	104<br>12.5%	217<br>26.1%	3<br>0.4%	0<br>0.0%	6<br>0.7%
G	275<br>0.4%	5<br>0.0%	262<br>0.4%	12<br>0.0%	342<br>0.5%	24<br>0.0%	56095<br>84.9%	1750<br>2.6%	887<br>1.3%	3847<br>5.8%	116<br>0.2%	30<br>0.0%	2436<br>3.7%
H	805<br>1.8%	2278<br>5.0%	353<br>0.8%	380<br>0.8%	2960<br>6.6%	18<br>0.0%	2678<br>5.9%	29697<br>65.8%	3437<br>7.6%	2341<br>5.2%	112<br>0.2%	24<br>0.1%	45<br>0.1%
I	136<br>0.4%	2268<br>6.2%	70<br>0.2%	45<br>0.1%	189<br>0.5%	5<br>0.0%	1301<br>3.6%	2523<br>6.9%	28577<br>78.3%	1351<br>3.7%	24<br>0.1%	0<br>0.0%	4<br>0.0%
J	338<br>0.5%	447<br>0.6%	22<br>0.0%	352<br>0.5%	2411<br>3.4%	22<br>0.0%	5419<br>7.7%	1934<br>2.8%	1922<br>2.7%	55105<br>78.7%	32<br>0.0%	477<br>0.7%	1499<br>2.1%
K	40<br>1.9%	214<br>10.4%	0<br>0.0%	15<br>0.7%	295<br>14.3%	2<br>0.1%	268<br>13.0%	440<br>21.3%	121<br>5.9%	174<br>8.4%	482<br>23.4%	0<br>0.0%	10<br>0.5%
L	76<br>0.8%	0<br>0.0%	0<br>0.0%	6<br>0.1%	14<br>0.2%	0<br>0.0%	52<br>0.6%	12<br>0.1%	0<br>0.0%	666<br>7.4%	0<br>0.0%	8063<br>89.9%	80<br>0.9%
M	0<br>0.0%	22<br>0.0%	0<br>0.0%	27<br>0.0%	30<br>0.0%	0<br>0.0%	1829<br>3.0%	67<br>0.1%	71<br>0.1%	1962<br>3.2%	5<br>0.0%	164<br>0.3%	56561<br>93.1%

A: Evergreen Needleleaf Forest, B: Evergreen Broadleaf Forest, C: Deciduous Needleleaf Forest, D: Deciduous Broadleaf Forest, E: Mixed Forest, F: Closed Shrubland, G: Open Shrubland, H: Woody Savanna, I: Savanna, J: Grassland, K: Wetland, L: Snow and Ice, M: Desert
