# Supplementary material for: Predicting dominant terrestrial biomes at a global scale using machine learning algorithms, climate variable indices, and extreme event indices
Source: PLoS One. 2026 Feb 26;21(2):e0324107. doi: 10.1371/journal.pone.0324107 (PMC12944746; doi:10.1371/journal.pone.0324107)
Supplement: S3 Table — The same dataset and evaluation as in S2 Table were used. (PDF) [file pone.0324107.s014.pdf]

S3 Table. Confusion matric for biome classification using the SVM model. The same dataset and evaluation as in S2 Table were used.

Predicted Class (columns); Actual Class (rows)

	A	B	C	D	E	F	G	H	I	J	K	L	M
A	7260<br>77.1%	0<br>0.0%	753<br>8.0%	153<br>1.6%	618<br>6.6%	0<br>0.0%	122<br>1.3%	284<br>3.0%	210<br>2.2%	17<br>0.2%	2<br>0.0%	0<br>0.0%	0<br>0.0%
B	1<br>0.0%	38716<br>88.0%	0<br>0.0%	161<br>0.4%	35<br>0.1%	1228<br>2.8%	3<br>0.0%	1287<br>2.9%	1784<br>4.1%	496<br>1.1%	274<br>0.6%	0<br>0.0%	2<br>0.0%
C	101<br>3.0%	0<br>0.0%	3186<br>95.1%	17<br>0.5%	40<br>1.2%	0<br>0.0%	50.1%<br>0.1%	0<br>0.0%	0<br>0.0%	0<br>0.0%	0<br>0.0%	0<br>0.0%	0<br>0.0%
D	31<br>0.4%	92<br>1.2%	0<br>0.0%	3923<br>49.3%	1971<br>24.8%	102<br>1.3%	19<br>0.2%	106<br>1.3%	1670<br>21.0%	37<br>0.5%	0<br>0.0%	0<br>0.0%	0<br>0.0%
E	11934<br>32.0%	290<br>0.8%	4079<br>11.1%	4093<br>11.0%	11402<br>30.6%	1223<br>3.3%	267<br>0.7%	2812<br>7.6%	485<br>1.3%	364<br>1.0%	279<br>0.7%	0<br>0.0%	15<br>0.0%
F	0<br>0.0%	83<br>10.0%	0<br>0.0%	18<br>2.2%	68<br>8.2%	241<br>29.0%	40<br>4.8%	10<br>1.2%	286<br>34.4%	24<br>2.9%	4<br>0.5%	0<br>0.0%	57<br>6.9%
G	2797<br>4.2%	35<br>0.1%	8188<br>12.4%	1161<br>1.8%	133<br>0.2%	2861<br>4.3%	30795<br>46.6%	139<br>0.2%	4506<br>6.8%	2184<br>3.3%	0<br>0.0%	3973<br>6.0%	9309<br>14.1%
H	4671<br>10.4%	11905<br>26.4%	4225<br>9.4%	2908<br>6.4%	937<br>2.1%	6485<br>14.4%	2133<br>4.7%	2117<br>4.7%	8798<br>19.5%	826<br>1.8%	55<br>0.1%	13<br>0.0%	55<br>0.1%
I	543<br>1.5%	12194<br>33.4%	1127<br>3.1%	415<br>1.1%	94<br>0.3%	5765<br>15.8%	823<br>2.3%	287<br>0.8%	15158<br>41.5%	66<br>0.2%	10<br>0.0%	10<br>0.0%	1<br>0.0%
J	6950<br>9.9%	1578<br>2.3%	1770<br>2.5%	4051<br>5.8%	8234<br>11.8%	6209<br>8.9%	19437<br>27.8%	1914<br>2.7%	4052<br>5.8%	6600<br>9.4%	98<br>0.1%	3827<br>5.5%	5260<br>7.5%
K	770<br>37.4%	476<br>23.1%	335<br>16.3%	56<br>2.7%	54<br>2.6%	67<br>3.3%	58<br>2.8%	43<br>2.1%	193<br>9.4%	0<br>0.0%	0<br>0.0%	0<br>0.0%	9<br>0.4%
L	361<br>4.0%	0<br>0.0%	0<br>0.0%	6<br>0.1%	48<br>0.5%	0<br>0.0%	279<br>3.1%	70<br>0.8%	0<br>0.0%	122<br>1.4%	100<br>1.1%	7983<br>89.0%	0<br>0.0%
M	169<br>0.3%	84<br>0.1%	0<br>0.0%	82<br>0.1%	50<br>0.1%	498<br>0.8%	4226<br>7.0%	10<br>0.0%	111<br>0.2%	174<br>0.3%	2<br>0.0%	817<br>1.3%	54515<br>89.8%

A: Evergreen Needleleaf Forest, B: Evergreen Broadleaf Forest, C: Deciduous Needleleaf Forest, D: Deciduous Broadleaf Forest, E: Mixed Forest, F: Closed Shrubland, G: Open Shrubland, H: Woody Savanna, I: Savanna, J: Grassland, K: Wetland, L: Snow and Ice, M: Desert
