# Supplementary material for: Predicting dominant terrestrial biomes at a global scale using machine learning algorithms, climate variable indices, and extreme event indices
Source: PLoS One. 2026 Feb 26;21(2):e0324107. doi: 10.1371/journal.pone.0324107 (PMC12944746; doi:10.1371/journal.pone.0324107)
Supplement: S4 Table — The same dataset and avaluation procedure as in S2 Table were used. (PDF) [file pone.0324107.s015.pdf]

S4 Table. Confusion matrix for biome classification using the CNN model. The same dataset and avaluation procedure as in S2 Table were used.

Predicted Class

Actal Class

	A	B	C	D	E	F	G	H	I	J	K	L	M
A	6032<br>64.0%	0<br>0.0%	4<br>0.0%	66<br>0.7%	1788<br>19.0%	0<br>0.0%	115<br>1.2%	796<br>8.4%	14<br>0.1%	575<br>6.1%	15<br>0.2%	25<br>0.3%	0<br>0.0%
B	0<br>0.0%	39085<br>88.7%	0<br>0.0%	37<br>0.1%	303<br>0.7%	0<br>0.0%	7<br>0.0%	2464<br>5.6%	1847<br>4.2%	323<br>0.7%	4<br>0.0%	0<br>0.0%	0<br>0.0%
C	4<br>0.1%	0<br>0.0%	2628<br>78.2%	14<br>0.4%	290<br>8.6%	0<br>0.0%	187<br>5.6%	208<br>6.2%	0<br>0.0%	26<br>0.8%	3<br>0.1%	0<br>0.0%	0<br>0.0%
D	3<br>0.0%	11<br>0.1%	0<br>0.0%	5045<br>63.9%	1774<br>22.5%	0<br>0.0%	76<br>1.0%	251<br>3.2%	306<br>3.9%	434<br>5.5%	0<br>0.0%	0<br>0.0%	0<br>0.0%
E	896<br>2.4%	279<br>0.7%	87<br>0.2%	1054<br>2.8%	30256<br>80.9%	1<br>0.0%	96<br>0.3%	2306<br>6.2%	42<br>0.1%	2369<br>6.3%	2<br>0.0%	2<br>0.0%	0<br>0.0%
F	0<br>0.0%	47<br>5.7%	0<br>0.0%	6<br>0.7%	57<br>6.9%	47<br>5.7%	250<br>30.1%	138<br>16.6%	114<br>13.7%	164<br>19.8%	0<br>0.0%	0<br>0.0%	7<br>0.8%
G	341<br>0.5%	6<br>0.0%	435<br>0.7%	16<br>0.0%	429<br>0.6%	10<br>0.0%	55385<br>83.9%	1692<br>2.6%	928<br>1.4%	4127<br>6.3%	26<br>0.0%	41<br>0.1%	2584<br>3.9%
H	766<br>1.7%	2795<br>6.2%	621<br>1.4%	584<br>1.3%	3408<br>7.6%	8<br>0.0%	2892<br>6.4%	27908<br>61.9%	3846<br>8.5%	2150<br>4.8%	16<br>0.0%	37<br>0.1%	39<br>0.1%
I	165<br>0.5%	2957<br>8.1%	74<br>0.2%	42<br>0.1%	201<br>0.6%	1<br>0.0%	1439<br>3.9%	3650<br>10.0%	26708<br>73.1%	1301<br>3.6%	2<br>0.0%	0<br>0.0%	0<br>0.0%
J	493<br>0.7%	565<br>0.8%	19<br>0.0%	395<br>0.6%	2841<br>4.1%	11<br>0.0%	5502<br>7.9%	2596<br>3.7%	2432<br>3.5%	52843<br>75.5%	1<br>0.0%	575<br>0.8%	1707<br>2.4%
K	74<br>3.6%	283<br>13.7%	0<br>0.0%	16<br>0.8%	327<br>15.9%	0<br>0.0%	467<br>22.7%	561<br>27.2%	93<br>4.5%	162<br>7.9%	74<br>3.6%	0<br>0.0%	3<br>0.1%
L	59<br>0.7%	0<br>0.0%	0<br>0.0%	8<br>0.1%	9<br>0.1%	0<br>0.0%	48<br>0.5%	17<br>0.2%	0<br>0.0%	565<br>6.3%	0<br>0.0%	8156<br>90.8%	118<br>1.3%
M	0<br>0.0%	37<br>0.1%	0<br>0.0%	34<br>0.1%	19<br>0.0%	0<br>0.0%	2176<br>3.6%	88<br>0.1%	64<br>0.1%	2148<br>3.5%	0<br>0.0%	128<br>0.2%	55886<br>92.3%

A: Evergreen Needleleaf Forest, B: Evergreen Broadleaf Forest, C: Deciduous Needleleaf Forest, D: Deciduous Broadleaf Forest, E: Mixed Forest, F: Closed Shrubland, G: Open Shrubland, H: Woody Savanna, I: Savanna, J: Grassland, K: Wetland, L: Snow and Ice, M: Desert
