# Supplementary material for: Predicting dominant terrestrial biomes at a global scale using machine learning algorithms, climate variable indices, and extreme event indices
Source: PLoS One. 2026 Feb 26;21(2):e0324107. doi: 10.1371/journal.pone.0324107 (PMC12944746; doi:10.1371/journal.pone.0324107)
Supplement: Attachment — Submitted filename: ReponseLetter.docx [file pone.0324107.s018.docx]

# Reviewer #1:

*> I thank the author for addressing my previous comments, and I am satisfied with the responses. The figures and tables have been improved, and the manuscript is now clearer and sound. I recommend acceptance.*

Response:

Thank you very much for your positive evaluation and supportive comments.

I sincerely appreciate your careful reading of the revised manuscript and your recognition of the improvements.

# Reviewer #4:

*> The author presents a study on modelling biome distribution at global scale using machine learning under present and future climate scenarios. This is the second time I read the manuscript, which has been largely revised, greatly improving its quality compared to the previous version. The author has made an effort to respond carefully to all comments. In particular, the overall research questions are stated more clearly and most of the methodological choices are now better justified.*

**Response:**

Thank you very much for your positive evaluation and supportive comments.

I sincerely appreciate your careful reading of the revised manuscript and your recognition of the improvements.

Below, I provide point-by-point responses to each of your comments.

## (1) Comment on L86-88.

*> I still question the relevance of retaining in the analysis grid cells that are highly impacted by human activities but do not reach 100%. The manuscript states that "grid cells partially affected by human activity were retained, on the assumption that the relative proportions of natural vegetation remain stable despite these changes. However, it has been shown that PNV maps disagree when human activities are high, suggesting that the proportion of remaining natural vegetation is not sufficiently informative in these grid cells. I therefore find the justification insufficient.*

**Response:**

In Methods (section "Biome data"), I added a brief explanation of the study aim-estimating PNV at 0.5°-and why grid cells with partial human influence still retain an informative dominant natural-vegetation signal at this scale. I also specified the exclusion of cells with 100% human cover and/or water and the corresponding exclusion ratio, and explicitly noted increased uncertainty in heavily impacted regions.

**[Inserted text (L102-109)]**

Because the aim of this study is to model potential natural vegetation (PNV), I retained grid cells that are only partially affected by human activity, on the premise that the dominant natural-vegetation signal remains informative at 0.5° resolution. By contrast, cells with 100% human cover and/or water were excluded, leaving 52,297 land grid cells (approximately 10-11% of land cells excluded, Antarctica removed). This choice minimizes spatial coverage bias while preserving information on the prevailing natural type. I acknowledge, however, that uncertainty in PNV estimation may increase in regions with strong human influence, and I interpret results in those areas with caution.

## (2) Comment on L93-94.

*> While the manuscript explains the importance of using a PNV map over maps derived from satellite imagery, and describes the product used, the choice of this specific PNV map for this study over others is insufficiently justified. The statement <<this dataset was selected because it is particularly well suited for global climate-vegetation modeling >> is not associated to any argument.*

**Response:**

In Methods, I added sentences giving practical reasons for selecting this PNV dataset (alignment with my analysis resolution, consistent processing with *AveI*/*CEI* and future projections, and improved comparability with related studies).

**[Inserted text (L94-101)]**

I used this PNV dataset for three practical reasons aligned with global climate-vegetation modelling at 0.5°: (i) the workflow-deriving the dominant natural class from MODIS/IGBP and resampling to ~50-km grids-matches my analysis grid; (ii) its climate inputs and projections are processed consistently with the BIOCLIM (*AveI*) and CLIMDEX (*CEI*) indices used here, with harmonized definitions and resolution for present data and CMIP5-based futures; and (iii) employing the same dataset as related machine-learning studies facilitates comparability without additional preprocessing.

## (3) Comment on L352-366.

*> The paragraph justifying the choice of these four specific ML algorithms and explaining their differences appears in the Discussion section. As it highlights one of the strengths of the study and illustrates well how the manuscript adresses the overall research questions, it would be more appropriate in the ‘Methods’ section under ‘Machine learning algorithms’.*

**Response:**

As suggested, I kept only the opening performance-summary sentence in the Discussion and moved the subsequent rationale and contrasts among the algorithms to Methods (Machine learning algorithms). I also added a one-sentence pointer in the Discussion directing readers to Methods; the substantive content itself was unchanged.

**[Inserted text (L382-384)]**

A concise rationale for selecting these four algorithms and their contrasting assumptions is provided in the Methods section ("Machine learning algorithms").

## (4) Comment on L392-402.

*> The manuscript recommends not including extreme climate data since it reduces robustness (here, consistency among models) under forecast climate conditions. However, the differences observed in predictions of the future state of vegetation also make it possible to define a universe of possibilities and therefore seem rather interesting. The fact that this extreme climate data improves test accuracy, even slightly, makes this observation even more interesting. The possibility that only one of these models accurately predicts the response of biome distribution to climate change remains plausible, although unprovable. The choice of whether to include or exclude this data therefore seems to me to depend on the research question, and would thus benefit from more discussion.*

**Response:**

To avoid redundancy, I deleted the original closing sentence of the paragraph and replaced it with three sentences that (i) define my operational use of robustness, (ii) attribute the loss of consistency to CEI variables with strong distributional shifts, and (iii) provide purpose-dependent guidance on when to include or exclude *CEI*.

**[Deleted L400-402 of the cleaned previous manuscript]**

Although consistency is used here as a proxy for robustness, it may also indicate a lack of sensitivity to novel or extreme conditions.

**[Inserted text (L419-427)]**

Here, "robustness" is operationalized as cross-algorithm consistency under future climates; while this proxy is useful for decision-making, it can also reflect reduced sensitivity to novel or extreme conditions. The observed loss of consistency was primarily driven by a small set of *CEI* variables whose distributions diverged strongly between training and projection conditions. In practice, whether to include *CEI* should follow the research question: (i) for a high-agreement baseline map under extrapolative climates, exclude *CEI*; (ii) to explore a broader possibility space or risk envelopes, include *CEI* as a supplementary input; and (iii) for species- or local-scale questions, extremes may contribute more and could be prioritized.
